# PEG-aspargase and DEP regimen combination therapy for refractory Epstein–Barr virus-associated hemophagocytic lymphohistiocytosis

**DOI:** 10.1186/s13045-016-0317-7

**Published:** 2016-09-09

**Authors:** Jingshi Wang, Yini Wang, Lin Wu, Jia Zhang, Wenyuan Lai, Zhao Wang

**Affiliations:** Department of Hematology, Beijing Friendship Hospital, Capital Medical University, Beijing, China

**Keywords:** PEG-aspargase, Epstein–Barr virus, Hemophagocytic lymphohistiocytosis

## Abstract

**Background:**

Epstein–Barr virus-associated hemophagocytic lymphohistiocytosis (EBV-HLH) is the most frequent subtype of secondary HLH triggered by infections. Previous studies have shown that ~30 % or more of patients with EBV-HLH do not respond to standard therapy. This study investigated the efficacy and safety profile of a modified DEP regimen in combination with PEG-aspargase (L-DEP) as a salvage therapy for refractory EBV-HLH.

**Methods:**

In this study from October 2014 to October 2015, 28 patients with refractory EBV-HLH received a L-DEP regimen at the Beijing Friendship Hospital, Capital Medical University. Treatment efficacy and adverse events were evaluated at 2 and 4 weeks after L-DEP treatment.

**Results:**

Median EBV-DNA concentrations before and 2 weeks after receiving the L-DEP regimen were 9.6 × 10^5^ (1.5 × 10^4^ − 1 × 10^9^) copies/mL and 2.2 × 10^5^ (3.8 × 10^2^ − 1.2 × 10^7^) copies/mL, respectively; the post-treatment values were significantly lower than that of the pretreatment (*P* = 0.048). Nine of the 28 study patients achieved complete response (CR) and 15 partial response (PR), resulting in an overall response rate of 85.7 % (CR+PR). Four patients who did not achieve response died within 4 weeks of receiving L-DEP. Thirteen of the 24 patients who achieved partial or complete response received subsequent allogenic hematopoietic stem cell transplantation (allo-HSCT). Ten of these 13 patients survived until 1 March 2016. The major adverse effects of the L-DEP regimen were high serum amylase concentrations, abnormal liver function, and coagulation disorders.

**Conclusions:**

This study suggests that L-DEP is a safe and effective salvage therapy prior to allo-HSCT for refractory EBV-HLH and increases the possibility of such patients receiving allo-HSCT. A prospective multicenter large-scale clinical trial that aims to validate the L-DEP regimen for refractory EBV-HLH is currently underway (ClinicalTrails.gov Identifier: NCT02631109).

## Background

Hemophagocytic lymphohistiocytosis (HLH) is a group of clinical syndromes characterized by fever, hepatosplenomegaly, pancytopenia, and hemophagocytic phenomena in the bone marrow, liver, spleen, and lymph tissue. HLH is divided into two categories: primary and acquired. Primary HLH is an autosomal recessive genetic disease, whereas acquired HLH is often associated with and caused by infections, malignant tumors, and autoimmune diseases. Among the infection-related forms of HLH, Epstein–Barr virus (EBV) infection-related HLH (EBV-HLH) is the most common [1], being particularly common in Asian countries. A previous analysis reported a 1-year overall survival (OS) of only 25.0 % for patients with EBV-HLH [2]. The main current first-line treatment for patients with EBV-HLH is the HLH-94 regimen [3], which comprises etoposide, dexamethasone, and cyclosporine A, with or without intrathecal injection of methotrexate, followed by allogeneic hematopoietic stem cell transplantation (allo-HSCT). Imashuku et al. [4] reported that the HLH-94 regimen improves the 43-month survival rate of EBV-HLH patients by 75.6 %. However, in a previous study of this regimen [3], approximately 30 % of patients with EBV-HLH had no response to therapy, most deaths occurring in the first few weeks after initiating treatment. In addition, the overall mortality rate was significantly higher in patients with active disease at the time of allo-HSCT (*P* = 0.014) [5]. There is currently no consensus on salvage therapy for patients with EBV-HLH who fail to respond to HLH-94/HLH-04 regimens. Identification of effective salvage therapies that can bridge the gap to allo-HSCT in patients with refractory EBV-HLH is of utmost importance and worthy of further research.

In our center, we have used liposomal doxorubicin, etoposide, and high-dose methylprednisolone (i.e., the DEP regimen) to treat EBV-HLH patients who have not achieved response on the HLH-94 regimen and achieved partial response (PR) or better efficacy in 72.7 % of such patients [6]. However, the duration of response after this regimen is relatively short and there is a significant risk of gastrointestinal bleeding. In this study, we modified the dosage and duration of methylprednisolone in the DEP regimen and combined it with PEG-aspargase (PEG-Asp) regimen (i.e., L-DEP regimen) as salvage therapy for refractory EBV-HLH and evaluated the efficacy and adverse drug reactions of this combination for treating EBV-HLH.

## Methods

### Patients and diagnostic criteria for refractory EBV-HLH

Eligibility criteria for this study were as follows: (1) meet HLH-04 diagnostic criteria [7], (2) high values for EBV-DNA copies in the peripheral blood or tissues or number of cells containing EBV-encoded small RNA (EBER) in the peripheral blood or tissues, (3) diagnosis of primary HLH excluded, (4) extranodal lymphoma excluded by positron emission tomography-computed tomography and repeated pathological examination of biopsy specimens, (5) treated with HLH-94 no less than 2 weeks before enrollment and did not achieve at least PR, and (6) did not have acute or chronic pancreatitis or active gastrointestinal bleeding and had a left ventricular ejection fraction of ≥50 % at the time of enrollment. In addition, all patients underwent bone marrow flow cytometry to detect monoclonal cells. The research protocol for this study was approved by the Ethics Committee of Beijing Friendship Hospital, Capital Medical University. All patients provided written informed consent before participating in the study.

EBV infection was confirmed by identifying significantly increased EBV-DNA copies in the peripheral blood or tissues or number of cells containing EBER in the peripheral blood or tissues [8]. In the absence of accepted diagnostic criteria, refractory HLH was defined according to previous research findings [9] and our clinical experience as failure to achieve at least PR according to an evaluation 2 weeks after receiving HLH-94 induction therapy.

### L-DEP regimen

**Fig. 1:**
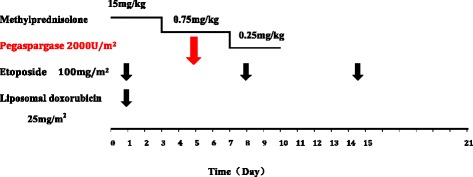
Protocol for L-DEP regimen

The L-DEP regimen used was as follows: PEG-aspargase 2000 U/m^2^ on day 5; liposomal doxorubicin (doxorubicin hydrochloride liposome injection) 25 mg/m^2^/day, day 1; etoposide 100 mg/m^2^/day on the first day of every week; and methylprednisolone 15 mg/kg/day for days 1 to 3, 0.75 mg/kg/day for days 4 to 7, and 0.25 mg/kg/day for days 8 to 10 (Fig. 1). Efficacy was evaluated 2 and 4 weeks after initiating L-DEP salvage therapy. Patients underwent allo-HSCT as soon as control of their HLH had been achieved. L-DEP regimen could be repeated after 3 weeks of salvage therapy in some patients who had not received further allo-HSCT for various reasons.

### Conditioning regimen, donors, and prevention of GVHD after allo-HSCT

The study patients received total body irradiation (TBI)/cyclophosphamide/etoposide (VP-16) conditioning (TBI, 4 Gy/day for days −8 to −7; etoposide 5 mg/kg/day for days −6 to −5; cyclophosphamide, 1.8 g/m^2^/day for days −4 to −3) or busulfan/cyclophosphamide/VP-16 conditioning (etoposide 5 mg/kg/day for days −9 to −8; busulfan 0.8 mg/kg, every 6 h for days −7 to −5; and cyclophosphamide, 1.8 g/m^2^/day for days −4 to −3).

High-resolution HLA typing was performed by polymerase chain reaction amplification with sequence-specific primers to determine HLA-A,-B, -Cw, -DRB1. and -DQ locus types. All donors underwent EBV-DNA and natural killer (NK) activity tests and were screened for the HLH-associated genes to exclude the possibility of carrying this disease.

All patients received cyclosporine A + methotrexate + anti-thymocyte globulin to prevent graft versus host disease (GVHD). Cyclosporine A, 3 mg/kg/day, was intravenously administered from day −8. Methotrexate (15 mg/m^2^/day) was administered on day +1 of the transplant, followed by 10 mg/m^2^/day on days +3, +5, and +11. Anti-thymocyte globulin, 3 mg/kg for patients with HLA-matched-related donors and 8 mg/kg for those with mismatched-related donors, was also administered.

### Evaluation criteria, observed indicators, and complications

The efficacy of L-DEP for the treatment of EBV-HLH was assessed according to the evaluation criteria proposed by Marsh et al. [9]. A complete response (CR) was defined as normalization of all quantifiable symptoms and laboratory markers of HLH, including values for soluble CD25, ferritin, triglyceride; hemoglobin; neutrophil and platelet counts; and alanine aminotransferase (ALT). A PR was defined as at least a 25 % improvement in two or more quantifiable symptoms and laboratory markers by 2 weeks following the L-DEP regimen as follows: soluble CD25 response 1.5-fold decreased; ferritin and triglyceride decreased at least 25 %; an increase by at least 100 % to >0.5 × 10^9^/L in patients with an initial neutrophil count of <0.5 × 10^9^/L; an increase by at least 100 % to >2.0 × 10^9^/L in patients with an initial neutrophil count of 0.5 to 2.0 × 10^9^/L; and decrease of at least 50 % in patients with initial ALT >400 U/L. Additionally, the subjects’ body temperature had to revert to normal ranges to diagnose either CR or PR. Failure to achieve PR was defined as no response.

Other observational indicators included EBV-DNA, bilirubin, and amylase. Adverse effects and complications, including pancreatitis, abnormal liver function, decline in fibrinogen, infection, hemorrhage and thrombosis, cardiac dysfunction, and adverse drug reactions were closely monitored during the treatment.

### Survival time

Patients received allo-HSCT as soon as control of their EBV-HLH had been achieved. Survival times were calculated from the date of L-DEP salvage therapy. All patients were followed up until death or 1 March 2016, whichever occurred first.

### Statistical analysis

SPSS 16.0 software (SPSS, Chicago, IL, USA) was used for statistical analysis. Because this was a small study, data that did not fit a normal distribution are presented as median and range. Comparisons between multiple samples and groups were performed using the Wilcoxon rank sum test. *P* < 0.05 was considered to denote a significant difference, and *P* < 0.01 was considered very significant. Kaplan–Meier survival curves were used to analyze the patients’ survival and the log-rank test to evaluate survival time.

## Results

### General patient characteristics

Twenty-eight patients complied with refractory EBV-HLH diagnostic criteria and received combined treatment of PEG-aspargase and DEP regimen (L-DEP regimen) at the Beijing Friendship Hospital, Capital Medical University (Beijing, China) from October 2014 to October 2015. The 28 patients with refractory EBV-HLH comprised including 22 male and six female patients (male to female ratio = 3.67:1) of a median age of 24 years (range 7–50 years), 23 of them (82.1 %) being ≥18 years old. The clinical features at initial presentation and at the time of diagnosis of refractoriness are presented in Table 1. According to flow cytometry, 15 patients had abnormal phenotypes of NK cells in their bone marrow; however, pathologic examination of bone marrow specimens did not support a diagnosis of lymphoma. These patients had previously received HLH-94 and one of them had received combined HLH-94 regimen and immunization with rituximab monoclonal antibody. The median time from initial diagnosis of EBV-HLH to initiating the L-DEP regimen was 4 weeks (range, 2–11 weeks); eight patients (28.6 %) had received the L-DEP regimen ≤4 weeks from diagnosis. The median time from the end of HLH-94 to initiating the L-DEP regimen was 2 weeks (range, 1–8 weeks).

**Table 1:** Clinical features of study patients at the time of initial diagnosis and time of identification of refractoriness

Clinical features	Initial diagnosis	refractoriness
	Number (*n* = 28)/percentage (%)	Number (*n* = 28)/percentage (%)
Fever	28/100.0	28/100.0
Neutrophils <1 × 10^9^/L	15/53.6	13/46.4
Hgb <90 g/L	16/57.1	18/64.3
PLT <100 × 10^9^/L	23/82.1	25/89.3
TG >3 mmol/L	17/60.7	18/64.3
Fgb <1.5 g/L	10/35.7	16/57.1
Ferritin ≥500 μg/L	21/75.0	25/89.3
ALT >40 U/L	24/85.7	24/85.7
TBiL >17.1 μmol/L	16/57.1	18/64.3
LDH >190 U/L	18/64.3	21/75.0
Splenomegaly	28/100.0	28/100.0
Hemophagocytosis	21/75.0	22/78.6
Decline of NK cell activity	17/60.7	18/64.3
Elevated soluble CD25	25/89.3	28/100.0
Positive EBV-DNA in whole blood	28/100.0	28/100.0
Positive EBER in lymph node/bone marrow	3/10.7	4/14.3

*Hgb* hemoglobin, *PLT* platelet, *Fgb* fibrinogen, *ALT* alanine aminotransferase, *TBiL* total bilirubin, *LDH* lactic dehydrogenase

### Changes in selected indicators and EBV-DNA before and after L-DEP regimen

**Fig. 2:**
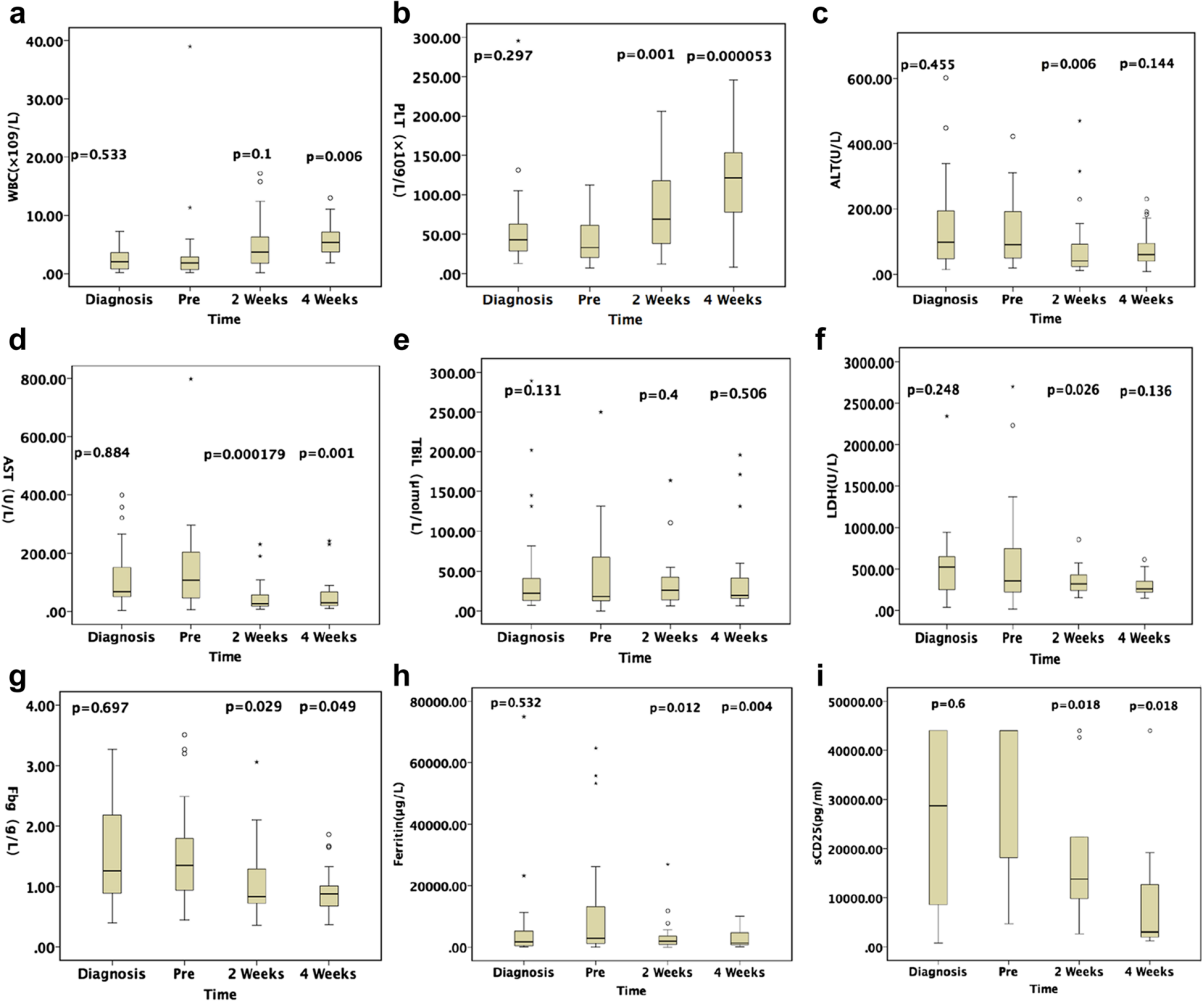
Changes in white blood cell counts (WBC) (**a**), platelet counts (**b**), alanine aminotransferase (ALT) concentrations (**c**), aspartate aminotransferase (AST) concentrations (**d**), total bilirubin (TBiL) concentrations (**e**), lactate dehydrogenase concentrations (**f**), fibrinogen (Fbg) concentrations (**g**), ferritin concentrations (**h**), and soluble CD25 concentrations (**i**) at the time of initial diagnosis of EBV-HLH, before, and 2 and 4 weeks after the L-DEP regimen

Nine variables were assessed at the time of initial diagnosis of EBV-HLH, before, and 2 and 4 weeks after the L-DEP regimen. These comprised white cell count, platelet count, alanine aminotransferase, aspartate aminotransferase, total bilirubin, lactate dehydrogenase, fibrinogen, ferritin, and soluble CD25 (Fig. 2). There were no significant statistical differences in these variables between the time of initial diagnosis of EBV-HLH and immediately before L-DEP salvage therapy. White blood cell counts were significantly higher 4 weeks after the L-DEP regimen than pretreatment (*P* = 0.006). Platelet counts were significantly higher at 2 and 4 weeks after the L-DEP regimen than pretreatment (*P* < 0.05), especially 4 weeks after the L-DEP regimen (*P* < 0.01). However, alanine aminotransferase, aspartate aminotransferase, and lactate dehydrogenase concentrations were significantly lower 2 weeks after the L-DEP regimen (*P* < 0.05); whereas total bilirubin values of the patients had not changed significantly at 2 and 4 weeks after the regimen (*P* > 0.05). Fibrinogen, ferritin, and soluble CD25 concentrations were lower at 2 and 4 weeks after the L-DEP regimen (*P* < 0.05).

Of the 28 patients with refractory EBV-HLH, the median EBV-DNA at the time of initial diagnosis of EBV-HLH, before and 2 weeks after L-DEP regimen were 1.1 × 10^6^ (1.4 × 10^3^–1 × 10^9^) copies/mL, 9.6 × 10^5^ (1.5 × 10^4^–1 × 10^9^) copies/mL, and 2.2 × 10^5^ (3.8 × 10^2^–1.2 × 10^7^) copies/mL, respectively. EBV-DNA values 2 weeks after L-DEP treatment were significantly lower than pretreatment (*P* = 0.048), whereas EBV-DNA values before L-DEP and at the time of initial diagnosis of EBV-HLH did not differ significantly (*P* = 0.427). EBV-DNA of three out of 28 patients was reduced to 0 copy/ml at four weeks after L-DEP regimen, while EBV-DNA of one patient reduced to 0 copy/ml at eight weeks after L-DEP regimen.

### Responses to L-DEP regimen and allo-HSCT

Nine of the 28 patients with refractory EBV-HLH achieved CR, 15 achieved PR, and four showed no evidence of response; thus, the overall response rate was 85.7 % (CR+PR). Six of the patients with CR and 11 of those with PR received a second cycle of L-DEP before further treatment because of donor or financial problems.

All nine patients with CR underwent allo-HSCT, one of them from an HLA-identical donor, whereas the remaining eight underwent haploid allo-HSCT. Of the 15 patients who achieved PR, four underwent haploid allo-HSCT, four did not undergo allo-HSCT for financial reasons, and the remaining seven relapsed before receiving allo-HSCT (4 to 6 weeks after L-DEP). Table 2 shows the clinical features of the 13 patients who received allo-HSCT after achieving PR or CR.

**Table 2:** Characteristics of patients who underwent allo-HSCT

Patient no.	Disease status	HSCT method	Donor	Conditioning regimen	GVHD prophylaxis	Adverse reactions	Outcome	Causes of death
1	CR	HLA 5/10 related donor	Sister	TBI/Cy/VP-16	CsA + MTX + ATG	Recurrence, acute GVHD I, sepsis	Died	Recurrence, sepsis
2	PR	HLA5/10 related donor	Brother	TBI/Cy/VP-16	CsA+MTX+ATG	Acute GVHD III	Survival	–
3	CR	HLA10/10 related donor	Sister	TBI/Cy/VP-16	CsA+MTX+ATG	Pulmonary infection, acute GVHD II	Survival	–
4	CR	HLA5/10 related donor	Brother	TBI/Cy/VP16	CsA+MTX+ATG	Recurrence, multiple organ failure	Died	Recurrence, multiple organ failure
5	CR	HLA5/10 related donor	Mother	TBI/Cy/VP-16	CsA+MTX+ATG	Acute GVHD I	Survival	–
6	PR	HLA5/10 related donor	Mother	Bu/Cy/VP-16	CsA+MTX+ATG	Pulmonary infection, gastrointestinal bleeding	Died	Pulmonary infection, gastrointestinal bleeding
7	CR	HLA5/10 related donor	Sister	Bu/Cy/VP-16	CsA+MTX+ATG	Pulmonary infection	Survival	–
8	CR	HLA5/10 related donor	Brother	TBI/Cy/VP-16	CsA+MTX+ATG	Acute GVHD I, gastrointestinal bleeding	Survival	–
9	PR	HLA5/10 related donor	Brother	TBI/Cy/VP-16	CsA+MTX+ATG	Acute GVHD I	Survival	–
10	CR	HLA5/10 related donor	Father	TBI/Cy/VP-16	CsA+MTX+ATG	Acute GVHD II, pulmonary infection	Survival	–
11	CR	HLA5/10 related donor	Father	TBI/Cy/VP-16	CsA+MTX+ATG	Acute GVHD III, hemorrhagic cystitis	Survival	–
12	CR	HLA5/10 related donor	Father	TBI/Cy/VP-16	CsA+MTX+ATG	–	Survival	–
13	PR	HLA5/10 related donor	Father	TBI/Cy/VP-16	CsA+MTX+ATG	Acute GVHD I, pulmonary infection	Survival	–

*allo-HSCT* allogeneic hematopoietic stem cell transplantation, *CR* complete response, *PR* partial response, *TBI* total body irradiation, *Cy* cyclophosphamide, *VP-16* etoposide, *Bu* busulfan, *CsA* cyclosporine A, *MTX* methotrexate, *ATG* anti-thymocyte globulin, *GVHD* graft versus host disease

### Survival time

**Fig. 3:**
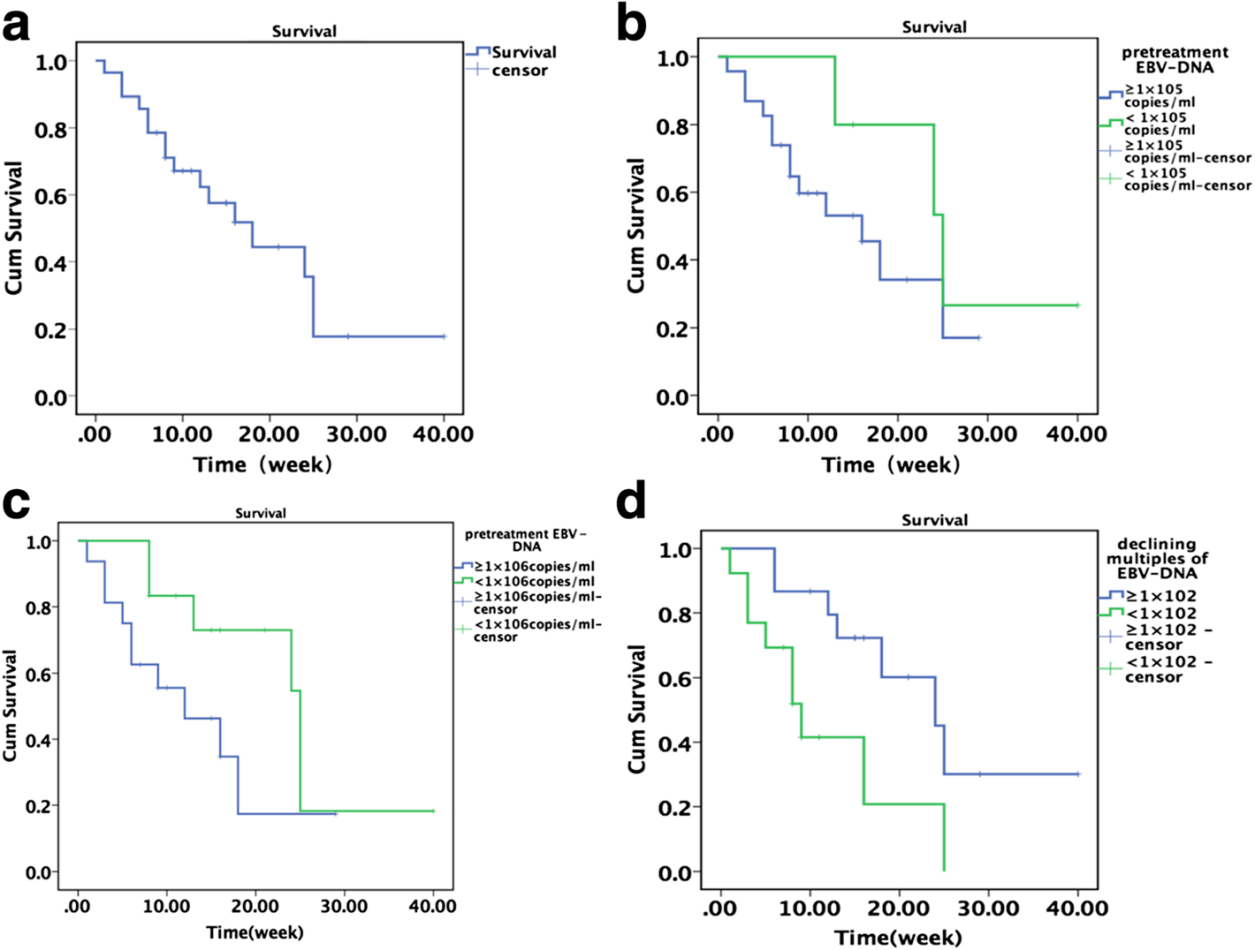
Survival of patients with refractory EBV-HLH (**a**). Relationship between the number of pretreatment EBV-DNA copies (1 × 10^5^ copies/mL) and survival (**b**). Relationship between the number of pretreatment EBV-DNA copies (1 × 10^6^ copies/mL) and survival (**c**). Relationship between declining multiples of EBV-DNA copies and survival after L-DEP regimen (**d**)

As of 1 March 2016, 14 of the 28 study patients EBV-HLH had survived and 14 had died; thus, the mortality was 50 % and the median survival 18 weeks (range 1–40 weeks, Fig. 3a). Three of the 13 patients who achieved PR or CR and underwent subsequent allo-HSCT died from EBV-HLH recurrence or transplant-related complications (Table 2). The four patients who achieved PR but did not undergo allo-HSCT for financial reasons maintained response for 4 to 20 weeks after L-DEP and died from relapse. Three of the seven patients who relapsed before receiving allo-HSCT died, and four patients are receiving a maintenance regimen at the time of publication. Four patients who did not achieve response died within 4 weeks of the L-DEP regimen.

Patients with EBV-DNA <1 × 10^5^ copies/mL before L-DEP treatment (*n* = 5) tended to survive longer than those with EBV-DNA ≥1 × 10^5^ copies/mL (*n* = 23); however, this difference was not statistically significant (*P* = 0.247, Fig. 3b). Similarly, patients with EBV-DNA <1 × 10^6^ copies/mL (*n* = 12) tended to survive longer than those with EBV-DNA ≥1 × 10^6^ copies/mL (*n* = 16); however, this difference was also not significant (*P* = 0.083, Fig. 3c). Nevertheless, patients who experienced a ≥1 × 10^2^-fold reduction in EBV-DNA copies after L-DEP treatment (*n* = 15) survived significantly longer than those with <1 × 10^2^-fold reduction in EBV-DNA copies (*n* = 13) (*P* = 0.018, Fig. 3d).

### Adverse reactions

#### Gastrointestinal disorders

Of the 28 patients with EBV-HLH treated with L-DEP, two developed grade 2 and one patient grade 3 pancreatitis. These three patients’ blood amylase concentrations reverted to normal levels after withholding food and water, acid-reduction, and somatostatin treatment. One patient had grade 2 oral mucositis and three patients grade 1–2 vomiting. One patient had grade 2 gastrointestinal bleeding; the remaining patients did not have grade ≥2 gastrointestinal bleeding.

#### Abnormal liver function

Of the 28 study patients, three developed increases in bilirubin concentrations <50 % of the baseline (pretreatment) values and two developed increases in bilirubin concentrations >50 % of baseline at 2–4 weeks after L-DEP treatment. Bilirubin concentrations decreased to below pretreatment values in the remaining patients. No patients experienced grade ≥3 hepatic failure.

#### Coagulation disorders

Fibrinogen concentrations were lower than 1.0 g/L in eight patients before salvage treatment. These patients received appropriate supportive therapies, including fresh frozen plasma and fibrinogen. Fibrinogen concentrations decreased to below pretreatment values in 20 of the 28 EBV-HLH patients receiving the L-DEP regimen; however, no patients developed grade ≥4 diffuse intravascular coagulation.

#### Infection

Pretreatment bacterial lung infections in 17 patients, pulmonary fungal infections in 10 patients, and tuberculosis infection in two patients did not worsen after effective anti-infective therapy. Twenty-seven patients required intravenous antibiotics for grade 3 bronchial infection; however, no patients had ≥grade 4 bronchial infection after therapy.

#### Bone marrow suppression and cytopenia

Before salvage treatment, neutrophils counts were lower than 1 × 10^9^/L in 13 patients, hemoglobin concentrations were lower than 90 g/L in 18 patients, and platelet counts were lower than 100 × 10^9^/L in 25 patients. Patients showed declines in neutrophil and platelet counts and hemoglobin concentrations approximately 1 week after the L-DEP regimen. However, patients who achieved response after L-DEP regimen had neutrophil and platelet counts and hemoglobin concentrations that were higher than pretreatment values 2 to 3 weeks after the L-DEP regimen. Two weeks after L-DEP treatment, neutrophil counts were lower than pretreatment values in four patients, as were hemoglobin concentrations in five patients and platelet counts in three patients. However, these values all decreased by less than 50 % of baseline values. Marrow toxicity from the L-DEP regimen was difficult to evaluate both because cytopenia is a major clinical feature of EBV-HLH and because counts are affected by granulocyte colony stimulating factor and blood transfusion.

## Discussion

HLH, an inflammatory cytokine storm caused by uncontrolled immune responses, is a rare life-threatening disease. HLH is divided into two major categories: primary and acquired. The latter is often caused by infections, malignant tumors, or autoimmune diseases. Infection-related HLH is mostly attributable to EBV infections, which account for approximately 70 % of infection-related cases [1]. EBV-HLH is common in Asian persons; however, it has also aroused attention in non-Asian countries in recent years. One study [10] has shown that adults with EBV-HLH have worse prognosis than children with this disease. However, there are no published large-scale clinical studies that adequately examine the prognoses of adults with HLH. In this study, 82.1 % of participants with EBV-HLH were adults. Thus, age may have some impact on the interpretation of the results of this study.

Immune system deficiencies are believed to be involved in the pathogenesis of EBV-HLH. It is well established that EBV and its antigens induce massive release of cytokines, thereby activating monocytes/macrophages and causing damage in multiple organs [11]. A series of Japanese-based studies have found that T cells and NK cells are targeted by EBV infection in patients with EBV-HLH [12, 13]. Fox et al. [13] reported abundant EBV genomes (12,900–1,816,550 genomes/10^6^ cells) in the circulating NK cells of such patients. Another study [14] also showed enhancement of proliferation of latent EBV-infected lymphocytes and that they contained new surface epitopes. Interestingly, etoposide (VP-16) inhibits EBV core epitope synthesis and has anti-EBV effects. One study [15] has shown that early administration of VP-16 and dexamethasone may reduce the mortality of EBV-HLH. Imashuku et al. used dexamethasone and VP-16 as the basic treatment regimen for 78 patients with EBV-HLH (73 of whom were ≤15 years old) and achieved a 43-month OS of 75.6 % [4]. Imashuku et al. [16] also reported enhancement of the long-term survival of patients with EBV-HLH treated with a regimen containing etoposide within 4 weeks of diagnosis; however, they found that treatment of patients with EBV-HLH with regimens containing cyclosporine made no significant difference to treatment efficacy or OS. Thus, the HLH-04 regimen is not significantly better than the HLH-94 regimen for treating patients with EBV-HLH. Although we found no significant difference in outcomes between 4-week HLH-04 and 4-week HLH-94 regimens in a previous study [2], patients with EBV-HLH who received these regimens had significantly better OS than patients who did not receive etoposide treatment. We therefore believe that etoposide-based immunochemotherapy improve the survival of patients with EBV-HLH. However, in a study by Henter et al. [3], >30 % of patients with EBV-HLH did not achieve response with the HLH-94 treatment regimen. To date, there is no established second-line treatment for patients with refractory HLH who do not respond to standard treatment regimens. Allo-HSCT-induced immune reconstitution in patients with EBV-HLH can result in them regaining the ability to remove EBV, thereby prolonging their survival and ultimately curing HLH. In one Japanese cohort, allo-HSCT resulted in an 85.7 % 10-year OS for patients with EBV-HLH [17]. However, allo-HSCT can cause an increase in transplant-related mortality in patients with poorly controlled EBV-HLH. In a previous study [2] in which we performed allo-HSCT in five patients with EBV-HLH, two of the five patients did not achieve response and died within approximately 3 months of diagnosis; one patient who did not achieve response after relapse died 7 months after diagnosis; and the remaining two patients, who received allo-HSCT while their disease was stable, survived. Thus, patients with EBV-HLH who do not achieve response before receiving allo-HSCT have a significantly reduced OS after transplantation, which is consistent with a previous report [5]. Hence, salvage treatment to stabilize the disease in patients with refractory EBV-HLH may be important in extending their survival after allo-HSCT.

Previous studies have demonstrated that anti-thymocyte globulin [18], tumor necrosis factor-alpha antagonists [19], anti-CD52 monoclonal antibody [9], anti-CD20 monoclonal antibody [20], and the DEP regimen [6] can be used as salvage therapy for refractory EBV-HLH. However, there is no established standard treatment for patients with EBV-HLH who fail to achieve response with the HLH-94 regimen. Beutel et al. [12] used rituximab (a CD20 monoclonal antibody) to label EBV-infected B lymphocytes and successfully treat B lymphocyte-related HLH. Imashuku et al. [21] postulated that EBV initially infects B cells and then continuously generates viral particles that further infect T or NK cells, suggesting that rituximab may be an effective treatment for EBV-infected T or NK cells. Based on the above theory, researchers have used rituximab to treat EBV-HLH; however, only retrospective studies and case reports are available. Chellapandian et al. [20] retrospectively analyzed the clinical information of 42 patients with EBV-HLH who received a rituximab monoclonal antibody treatment regimen. Rituximab-containing regimens appeared well tolerated and improved clinical status in 43 % of patients. However, no large follow-up studies of rituximab therapy in patients with EBV-HLH have been reported since 2013. In the study, one patient did not achieve response after a combination of rituximab monoclonal antibody and the HLH-94 regimen and further received the L-DEP regimen to achieve CR, which was followed by allo-HSCT. In our previous study, we used liposomal doxorubicin, etoposide, and high-dose methylprednisolone (i.e., the DEP regimen) to treat patients with EBV-HLH who did not achieve response after HLH-94; 72.7 % of those patients achieved PR or better treatment outcomes [6]. However, the duration of the consecutive treatment effect of the DEP regimen is relatively short and this regimen carries a significant risk of gastrointestinal bleeding. In this study, we modified the dosage and duration of methylprednisolone in the DEP regimen and combined it with PEG-aspargase (i.e., the L-DEP regimen) as salvage therapy for refractory EBV-HLH; this combination increased the overall response rate to 85.7 % and enabled more patients to receive allo-HSCT, including all those who achieved CR (100 %). Thirteen of the 28 patients with EBV-HLH received allo-HSCT. Only one patient received allo-HSCT from an HLA-identical donor and the remaining 12 patients underwent haploid allo-HSCT, which solved the limitation of donor problem due to “one-child” policy in China. Ten of these 13 patients survived (76.9 %) after allo-HSCT. Fifteen of the 28 study patients did not receive allo-HSCT for various reasons. Despite four (26.7 %) of the 15 patients are continuously receiving maintenance treatment at the time of publication, the remaining 11 patients do not survive.

L-DEP is a modified regimen in which PEG-aspargase is combined with the DEP regimen. PEG-aspargase, a new asparaginase preparation with a chemical coupling of modification in polyethylene glycol, not only retains the biological activity of L-asparaginase but also reduces its immunogenicity and its half-life from 20 h to 5.5 days, which may explain the improved treatment efficacy achieved by combining the DEP regimen with PEG-aspargase. Ando et al. [22] have reported that L-asparaginase induces in vitro apoptosis in NK lymphoma cells. In the present study, 15 of the 28 patients (53.6 %) demonstrated abnormal phenotypes of NK cells in the bone marrow as assessed by flow cytometry. In addition, these patients had significantly fewer EBV-DNA copies 2 weeks after L-DEP regimen than pretreatment (*P* = 0.048), EBV-DNA being undetectable after L-DEP regimen in four patients. We speculate that PEG-aspargase targets EBV-infected target cells, including T and NK cells, because these cells may not be able to synthesize L-asparagine themselves [22]. After entering cells, PEG-aspargase induces hydrolysis of L-asparagine, thus preventing target cells without L-asparagine (essential amino acids for protein syntheses) from synthesizing the corresponding proteins, ultimately inhibiting cellular proliferation and resulting in decline in EBV-DNA. An in vitro study by Jinta et al. [23] demonstrated that L-asparaginase dose-dependently reduces the number of EBV-positive T and NK cells; while not affecting the peripheral blood mononuclear cells of normal donors, suggesting that L-asparaginase inhibits the proliferation of EBV-positive T cells and NK tumor cells. A previous study [24] showed that high viral load of EBV-DNA may be a risk factor for poor outcomes. The patients less than 1 × 10^3^ copies/mL of EBVDNA showed a significantly higher clinical response and longer overall survival than those with high viral load of EBV-DNA. Another study [2] has shown that EBV-negativity is an independent risk factor for prognosis. Patients with EBV-negative conversion have a significantly better OS than patients who are continuously EBV-positive. During L-DEP treatment, the clinical status of patients with EBV-HLH improves, usually accompanied by reduction in EBV-DNA copies or conversion to EBV-DNA negativity. EBV replication is greater in patients with EBV-HLH with more serious disease manifestations. In this study, prognosis was not significantly associated with numbers of EBV-DNA copies before the L-DEP regimen (*P* > 0.05). However, reduction in EBV-DNA copies was associated with prognosis. After L-DEP treatment, reduction of ≥1 × 10^2^-fold in EBV-DNA copies was significantly associated with prolonged survival time (*P* = 0.018).

The DEP regimen is associated with increased risk of bleeding, especially from the gastrointestinal tract [6]. We therefore modified the DEP regimen by retaining high-dose corticosteroid therapy to suppress the inflammatory cytokine storm and rapidly control the disease. However, we rapidly decreased the dose of methylprednisolone and stopped it within 10 days, thus reducing the adverse effects of long-term and high-dose glucocorticoid therapy. In this study, no patient developed ≥grade 3 gastrointestinal bleeding. The major adverse effects of PEG-aspargase are liver dysfunction, decreased fibrinogen concentrations, and pancreatitis. In this study, no significant improvements in ALT or lactate dehydrogenase concentrations were found in the study patients in the fourth week after L-DEP treatment. In addition, total bilirubin concentrations 2 and 4 weeks after treatment did not differ significantly from pretreatment values (*P* > 0.05). However, other HLH indicators improved compared with pretreatment values, suggesting that impairment in liver function was caused by PEG-aspargase, especially in the five patients with higher bilirubin concentrations 2 and 4 weeks after treatment than pretreatment. However, this liver dysfunction was alleviated by liver protection therapy. In this study, fibrinogen concentrations were significantly lower 2 and 4 weeks after treatment than pretreatment in the study patients (*P* < 0.05), suggesting that this change was associated with coagulation disorders caused by PEG-aspargase. Supplementary human fibrinogen and fresh frozen plasma therapy prevented any serious bleeding in this cohort. Three patients had increased serum amylase concentrations after treatment, one of whom developed abdominal pain and radiographic changes. After somatostatin therapy, these patients’ amylase concentration reverted to normal values. In addition, we found that a low-fat diet after PEG-aspargase treatment appeared to reduce the incidence of adverse reactions.

## Conclusions

In conclusion, our findings suggest that the L-DEP regimen is a safe and effective salvage therapy prior to allo-HSCT for refractory EBV-HLH and enables more patients to undergo allo-HSCT. Although this was a small study, we have provided some clinical evidence for the efficacy of the L-DEP regimen in patients with refractory EBV-HLH. Our current prospective multicenter and large-scale clinical trial will further assess the L-DEP regimen for refractory EBV-HLH (ClinicalTrails.gov Identifier: NCT02631109).

## Abbreviations

Allo-HSCT: Allogeneic hematopoietic stem cell transplantation
ALT: Alanine aminotransferase
CR: Complete response
DEP regimen: Doxorubicin hydrochloride liposome, etoposide, and methylprednisolone
EBER: EBV-encoded small RNA
EBV: Epstein–Barr virus
GVHD: Graft versus host disease
HLH: Hemophagocytic lymphohistiocytosis
L-DEP regimen: PEG-aspargase plus DEP regimen
NK: Natural killer
OS: Overall survival
PR: Partial response
TBI: Total body irradiation
VP-16: Etoposide
